# Seasonal Variation in Essential Oil Composition and Yield of *Rhus aromatica* Aiton (Fragrant Sumac) and a Preliminary Comparison with *Rhus trilobata*

**DOI:** 10.3390/molecules30244722

**Published:** 2025-12-10

**Authors:** Tanner B. Beck, Prabodh Satyal, Michael C. Rotter, William N. Setzer, Ambika Poudel, Joseph E. Hilton

**Affiliations:** 1 doTERRA International, LLC, 389 S 1300 W, Pleasant Grove, UT 84062, USA; beck.tanner.byron@gmail.com (T.B.B.); apoudel@aromaticplant.org (A.P.); jhilton@doterra.com (J.E.H.); 2 Department of Biology, Utah Valley University, Orem, UT 84058, USA; mrotter@uvu.edu; 3 Aromatic Plant Research Center, 230 N 1200 E, Suite 100, Lehi, UT 84043, USA; setzerw@uah.edu

**Keywords:** *Rhus aromatica*, *Rhus trilobata*, essential oils, fragrant sumac, seasonal variation, plant drying methods, chiral, limonene

## Abstract

*Rhus* is a genus of shrubs that produces strong aromatic essential oils. These oils have numerous applications, including treating urinary infections, flavoring food, and alleviating gastrointestinal ailments. We conducted an experiment to track the essential oil yield and composition of two Rhus species, *Rhus aromatica* Aiton and *Rhus trilobata* Nutt., over the course of the growing season. The essential oil yield and composition were examined over four weeks from May to June 2023. We found that higher ambient temperature lowered the total yield of essential oils. Yield decreased in both species over the course of the experiment, with a yield of 0.21% in week 1 and 0.07% in week 4. The composition of oils changed as the outside temperature increased. Some of the lighter hydrocarbons, such as limonene, decreased, while other heavier hydrocarbons, including β-caryophyllene and germacrene D, increased as the outside temperature rose. Limonene was also shown to be the main component of *R. aromatica* essential oil at an average of 86.2% area, with >98% of that being the enantiomer D-limonene. Additionally, the effect of different drying methods on the yield and composition of the essential oils was investigated. The results show that when drying the plant material in the oven, a greater yield of 0.31% is obtained, whereas the shade- and sun-dry methods yield 0.09% and 0.02%, respectively. The main component of *R. trilobata* was α-pinene compared to the D-limonene in *R. aromatica*. This study helps to understand more of the correlation between outside temperature and essential yield/composition in the species *R. aromatica*, particularly during the May–June sampling period. To compare two *Rhus* species that get commonly grouped together as the same species, SPME Headspace was used to determine that the main component of *R. trilobata* was α-pinene compared to D-limonene being the main component in *R. aromatica*.

## 1. Introduction

Plant phytochemicals, chemical compounds synthesized by plants, have been well studied for their important ecological roles [1,2] evolutionary context [3,4], and use by humans [5]. The controls of the content, quality, and quantity of these phytochemicals are dictated by multiple inputs and at various scales [6,7]. Within groups of plants, and even within individual species, phytochemicals can vary over both space and time [8,9]. Understanding this diversity of variation in phytochemistry is a major goal for both elucidating the evolutionary ecological context of plants as well as for humans who take advantage of these compounds.

One of the prominent predictors of plant phytochemistry is the phenological patterns that plants follow in synthesizing these compounds [9]. Changes in phytochemicals in plants can fluctuate daily [10,11] as well as change seasonally [12]. As with any phytochemical composition, these phenological patterns can influence the content [13], quality [14], and quantity [15] of the phytochemicals that are produced by a plant. These daily and seasonal fluctuations are typically controlled by patterns such as water availability [16], temperature [17], solar availability [18], nutrient fluctuations [19], and/or biological responses [20].

The mechanisms regulating phytochemicals that are triggered by these biotic and abiotic cues are controlled by changes to genetically based traits (i.e., selection) and through tweaks to biochemical pathways. Between species, selection on biochemical pathways is likely the central point of divergence between related species [21]. For instance, cultivars of hemp and cannabis breeding have taken place over generations to emphasize particular biochemical pathways that have resulted in the production of particular phytochemicals [22]. Within a species phytochemicals vary largely by responses within (not between) biochemical pathways [23]. In *Teucrium arduini,* variation in phytocompounds can be a response to local environmental conditions [24]; this is likely related to cues that trigger changes in local biochemical pathways [25].

Because the ability to predict phytochemical concentration for specific uses is important from both ethnopharmacological and commercial perspectives, many cultures have developed patterns of plant use that take advantage of this phenology [26]. For example, traditional plants used for their antimicrobial properties in South Africa show distinct seasonal fluctuations in tannin and flavonoid levels [27], while *Artemisia dracunculus* is known to produce higher quantities of essential oil when water deficient [28]. In Uganda, rural communities will typically collect the important socio-medicinal plant *Moringa oleifera* only in the dry seasons in order to secure plants that have desired phytochemical levels [29]. In commercial applications of plant phytochemicals, timing and processing of plants are important considerations in the content, quality, and quantity of extractable products [30,31]. 

The genus *Rhus* (Anacardiaceae) comprises over 250 species of deciduous trees and shrubs, distributed worldwide. North American indigenous people have used them as dyes [32] and as medicines [33,34,35]. They have also been used medicinally and as a flavoring in Iran [36]. Of the genus, *Rhus aromatica* Aiton and *Rhus trilobata* Nutt. Both have had a prominent place in North American herbology [37].

*Rhus trilobata* and *R. aromatica* both have trifoliate leaves and are native to North America. *R. trilobata* is native to the western region of North America and parts of western Canada and Mexico, and *R. aromatica* occupies areas east of the Great Plains. *Rhus trilobata* generally has smaller flowers and fruits, as well as more distinctly lobed terminal leaves; however, it is challenging to use these characteristics to differentiate the two species. The best way to distinguish them is by geographical distribution [38]. Herbarium specimens have been collected and submitted to the UVSC Herbarium.

Both plants are used for their essential oils rich in the main component of D-Limonene. D-limonene is a monocyclic monoterpene that is abundantly found in citrus plants, such as lemons, oranges, limes, and grapefruits [39]. Limonene has a pleasant orange-like odor and is a colorless oil [40]. Some of the applications of D-limonene include having antitumor and anticancer properties, as it promotes apoptosis in certain cancer cells [41], as well as antioxidant, antidiabetic, and anti-inflammatory activity [39].

In this study, we investigated the seasonal variation in essential oil composition and yield in *R. aromatica*. We also wanted to compare this variation and yield to the closely related *R. trilobata.* Our goal was to identify the most efficient and productive time and post-harvest drying to extract the essential oils of these important ethnomedicinal plants for better future use.

## 2. Results

### 2.1. Essential Oil Yields Based on the Time of Year Collected

*Rhus aromatica* essential oils were shown to decrease in yield as temperature increased in the area where the plant material was collected. At an average temperature of 21 °C, the highest average temperature, we found that essential oil yields were the lowest, at an average of 0.06%. In the coldest average temperature of 15 °C, we found that essential oil yields were the highest at 0.21% (Table 1).

During the first week, we achieved a crude yield of 0.21% (Week 1) and 0.35% for the flowers (Table 1). After the first week, the yields dropped to 0.15% (Week 2) and then decreased to below 0.10% for the remainder of the experiment. This drop was paired with a corresponding increase in temperature over the observed weeks (Table 2).

### 2.2. Essential Oil Composition Based on the Time of Year Collected

The essential oils from each distillation were subjected to detailed GC-MS, GC-FID, and GC Chiral analyses to determine the compositions of each oil based on the week that it was collected. Lighter hydrocarbons, such as limonene and α-pinene, were shown to decrease as the temperatures rose from May to July in 2023. In contrast, other compounds, such as β-caryophyllene and germacrene D, showed a trending increase from the same months (Table 3).

Aerial parts (leaves, flowers and stems) were collected individually for two different distillations (neither distillation produced enough oil to run through GC-FID, so the hydrosol was compared as shown in (Table 4). 

### 2.3. Hydrosol Composition and Trends

The hydrosol compositions exhibited significant variations; however, certain compounds were consistently identified across the GC-MS reports. Terpinen-4-ol, α-terpineol, *epi*-α-cadinol, *epi*-α-muurolol, and α-cadinol are some of the most consistent compounds shown in the hydrosols that were tested. α-Cadinol was the main component of the *R. aromatica* and *R. trilobata* hydrosols. Because alcohols are more soluble in water than hydrocarbons, the alcohols shown here are more likely to show up in the hydrosol instead of staying in the essential oils during distillation, which is the reason for the high concentrations of these compounds in the hydrosols (Table 4). Because the hydrosol results varied significantly, it is challenging to identify an exact trend of compounds in relation to seasonal variation or drying methods. Because hydrosols contain the hydrophilic fraction of the distillate, higher levels of water-soluble compounds are expected. Solvent extraction and distillation heat may also introduce variability, which can explain elevated values such as octanoic acid.

### 2.4. Effect of Drying Method on Essential Oil Yield and Composition

When drying the plant material, the results show that the oven-drying method yielded more essential oil than the sun- or shade-drying methods. When drying the plant material in an oven, we obtained an essential oil yield of 0.31%, whereas the sun- and shade-dried methods yielded 0.09% and 0.02%, respectively (Table 1). In the oven- and shade-drying methods, we observed that the limonene content in the essential oils decreased compared to the freshly distilled material, with an average area of 78.6% and 86.2%, respectively (Table 3).

Each drying method was found to increase the area percentage of sesquiterpenes, including β-caryophyllene and germacrene D, which are heavier hydrocarbons compared to limonene. This may be caused by the high boiling point that sesquiterpenes possess, causing these molecules to increase in concentration as the temperature rises in the plants. We observe that the oven-drying method increases the germacrene D levels to 5.4%, compared to the 4.3% achieved by the shade-drying method (Table 3). Moisture content was not measured for the dried samples, which limits direct comparison with the fresh-weight yields reported for the weekly harvests.

### 2.5. Chemical Composition of Rhus aromatica Flower

Compared to the rest of the experiments performed on mostly leaf material, the distillation composed entirely of *R. aromatica* flower was different in that the limonene content was down to around 76.5% area, and the α-pinene content was more than that of the leaves at 6.9% compared to around 1% area (Table 3). It was expected that the flower of the plant would show different chemistry than the leaves and stems of the plant.

### 2.6. Chemical Difference Between Rhus aromatica and Rhus trilobata Using SPME Headspace

As demonstrated throughout this study, limonene is the primary compound of *R. aromatica*, as confirmed by SPME headspace analysis. SPME Headspace analysis showed that limonene remains the primary compound of *R. aromatica*. Because the plants are so similar, and since some taxonomists categorize *R. trilobata* as a subspecies of *R. aromatica*, it is assumed that this would also be the case for *R. trilobata*. However, the reports show that the main component in *R. trilobata* was α-pinene (22.3% area), and only 2.3% of the area was limonene. Sesquiterpenes such as germacrene D and β-caryophyllene are also a lot higher in *R. trilobata* compared to *R. aromatica* (Table 5).

SPME headspace analysis was carried out only on a single representative sampling rather than across all harvest weeks because this experiment was intended as a qualitative, exploratory comparison of volatile profiles, not as part of the seasonal quantitative dataset. GC-MS, GC-FID, and chiral GC were therefore used for all week-to-week comparisons.

With the use of GC Chiral, the enantiomer D-limonene was found to be the main component of the essential oils at >98% of the total limonene content, with <2% being the L-limonene enantiomer (Table 6). This can explain its “lemony scent” and a lot of its medicinal properties as well.

## 3. Discussion

Our results found variation in the phytochemical concentration of *R. aromatica* throughout the growing season. Although plant biomass increased over the course of our study, the essential oil yield dropped by around 50% (Table 3). This change is not all that unexpected, as many plants contain their highest levels of phytochemical production during times of early development and growth [9]. In addition, as the weeks progressed, we also saw a decline in yield that was associated with increasing average temperatures (Table 2). This could suggest that *R. aromatica* shifts resources and allocation within its biomass to either buffer itself from the increase in temperature [42] or that during plant phenology, there is a gradual reduction in these phytocompounds. It is important to point out that both ideas are not mutually exclusive [43].

Post-harvest drying was also an important factor in phytochemical extraction (Table 3). Notably, several phytochemicals increased the area percentage of sesquiterpenes. This may be caused by the high boiling point that sesquiterpenes possess, causing these molecules to increase in concentration as the temperature rises in the plants [44]. Post-processing material is a crucial aspect of extractions, particularly for specific compounds of interest. This is demonstrated in the traditional and contemporary harvesting and use of many plants for their compounds [45]. For instance, in Africa, *Vernonia amygdalina* must be rapidly dried at relatively high temperatures to preserve the most important phytochemical compounds in the plant [46]. Likewise, in our study system, quick drying with an oven instead of a longer process outside (in the sun or shade) would likely best preserve and concentrate the target phytocompounds. This is likely due to the rapid removal of moisture, which prevents the enzymatic breakdown of compounds [47]. Please note that these drying methods were performed in July using material different from that collected in May and June, separating these results from our seasonal variation effects results.

The monoterpene D-Limonene stands out as the primary compound found in *Rhus aromatica*. When we compare this to common citrus essential oils—specifically those derived from *Citrus sinensis* and *Citrus grandis*—limonene content often sits between 63% and 89%. Notably, the (+)-enantiomer in citrus peel oils grown in Nepal can achieve an impressive purity of up to 99.4%, a figure established by Bhandari et al. in 2021 [48].

Interestingly, our *Rhus aromatica* samples demonstrate a comparably high enantiomeric purity. What sets the *Rhus* samples apart, however, is their significantly greater stability across different seasons. Citrus oils, conversely, frequently experience fluctuations in both limonene levels and chiral ratios, which are sensitive to variables like altitude, fruit maturity, environmental temperature, and other factors. The consistent maintenance of a high proportion of (+)-limonene in *Rhus aromatica* strongly suggests a greater influence of genetic control and a much lower sensitivity to environmental variations than what we typically see across commercial citrus chemotypes.

We found significant differences between *R. aromatica* and *R. trilobata*, although they are very closely related (and once assumed to be the same species [38]). Phytochemical signatures of plants are typically seen as a great way to distinguish species [49] and infraspecific ranks [50]. These differences often reflect the consequences of differences in selection pressures. For these two allopatric species, selection pressures differ, likely not just from climate alone [51] but also from biotic selections [52]. These changes can happen very rapidly; in the case of *Erythranthe guttata*, separation of just over a century has caused divergence in phytochemistry between non-native plants and native plants that rivals the differences between genetic clades of native plants [53]. In the case of these two species of *Rhus,* the continental-scale divergence would easily predict our observed outcomes. Mass, fiber type, extraction time or temperature were not standardized in this experiment comparing the two species using the SPME Headspace.

This work performed on the essential oils of *R. aromatica* provides new information on its chemical composition, drying methods, and species differentiation. The results have indicated that the essential oil yields and the main component, D-limonene, are more prevalent when the plant is harvested at cooler temperatures in its environment, thus preserving the main compounds and achieving a higher essential oil yield. It is best to harvest the plant for its essential oils earlier in the spring to maximize the essential oil yield and D-limonene content and to use the medicinal properties. Waiting to harvest them until later in the season could risk losing important compounds that give the plant its medicinal properties. This species and its essential oils have potential for medicinal use in the future, and further research is necessary to fully understand their potential.

## 4. Materials and Methods

### 4.1. Rhus aromatica and Rhus trilobata Plant Material

*Rhus aromatica* Aiton was collected every eight days (Week 3 did not produce any oil, so the experiment was re-conducted on 14 May 2023 and replaced “Week 3” results) on doTERRA International, LLC (Limited Liability Company) campus grounds in Pleasant Grove, Utah 84062, USA (40°21′26.3″ N 111°45′27.1″ W, 1373 m elevation), starting on 22 May 2023 in the mornings from 9 to 11 am, and ending on 16 June 2023 on week 4. The aerial parts of the plant, including the flowers, leaves, and some of the stem, were collected. It was collected from various bushes of the same species across the doTERRA campus grounds. 100–300 g of material were collected each time. We pooled these results to show the trends. The flower and dried plant materials of *R. aromatica* were also tested separately for essential oil yield and chemistry.

*Rhus trilobata* Nutt. was collected 16 June 2023, at the Spring Creek Canyon Trail in Springville, Utah 84663, USA (40°09′48.1″ N 111°34′02.8″ W, 1573 m elevation). 

Sampling times were chosen to represent early, mid, and late stages of the growing season. Week 1 corresponded to early spring flush, Week 2 to mid-season vegetative growth, Week 3 to late-season leaf maturation, and Week 4 to the beginning of senescence. Not all plant organs were available at every time point. For example, flowers and young shoots were present only during early-season collections, while later collections contained mature leaves and stems without flowers. We have clarified this here because the presence or absence of specific organs may explain part of the compositional differences observed among weekly distillations.

### 4.2. Extraction and Analysis of Essential Oils

Plant material was added into a steam distillation (Clevenger-type apparatus) to extract the oils and was run for 4–6 h for every distillation. The essential oils were separated from the hydrosol and stored at 4 °C until use. Seasonal yields (Weeks 1–4) are reported on a fresh-weight basis, while drying method yields are expressed on a dry-weight basis. These values are not directly comparable.

Essential oil samples were analyzed on a GC–MS-QP2010 Ultra system (Shimadzu, Columbia, MD, USA). The instrument was operated in electron impact (EI) mode at 70 eV with a scan range of *m/z* 40–400 and a scan rate of 3.99 scans/s. Separation was achieved on a ZB-5-ms fused-silica capillary column ((5% phenyl)–polydimethylsiloxane stationary phase, 60 m × 0.25 mm i.d., 0.25 μm film thickness). Helium was used as the carrier gas at a constant flow of 1.0 mL/min. The injector temperature was set to 260 °C and the MS source at 260 °C. The oven temperature program was 40 °C, increased at 2 °C/min to 260 °C. Samples were diluted to 5% *w*/*v* in dichloromethane, and 0.1 μL was injected using a 30:1 split ratio.

Compounds were identified using a combination of mass spectral matching, retention indices, and authenticated standards. Mass spectra were compared against the SatSet Essential Oil Library [54] as well as the NIST and FFNSC spectral databases. Retention indices (RI) were calculated relative to a homologous series of *n*-alkanes (C8–C30) under identical chromatographic conditions. Identifications were confirmed by comparison with published literature values and available authenticated reference standards.

Quantitative analyses were performed using a GC-2010 Plus (Shimadzu), Columbus MD, USA, equipped with a flame ionization detector and the same ZB-5-ms column and oven program described above.

Injector and detector temperatures were 260 °C and 280 °C, respectively. Peak areas were integrated using Shimadzu LabSolutions software ver 5.11 conducted as described previously in [55]. Compounds were identified by using retention indices and GC-MS information.

Enantiomeric distributions were determined using a chiral GC column (e.g., Rt-BetaDEXsm, 30 m × 0.25 mm × 0.25 μm). The oven was programmed from 40 °C (5 min) to 200 °C at 2 °C/min. Helium was used as the carrier gas (1.0 mL/min), with injector and detector temperatures at 230 °C and 250 °C. Enantiomers were identified by comparing retention times to authenticated chiral standards purchased from Sigma Aldrich, Saint Louis, MO, USA.

For qualitative volatile analysis, a PDMS SPME fiber (100 μm) was exposed to the headspace of crushed leaf material in water for 30 min at room temperature. Desorption occurred in splitless mode at 250 °C for 1 min. This method was used only for exploratory profiling and interspecific comparison.

### 4.3. Rhus aromatica and Rhus trilobata Hydrosol Extraction Method

The hydrosol was collected from each steam distillation, and GC-MS analysis was used to identify the compounds present in the hydrosol after extraction. Extraction of the compounds from the hydrosol was performed using dichloromethane (DCM) purchased from Sigma Aldrich, Saint Louis, MO, USA. The hydrosol (100 mL) was extracted with DCM (10 mL) three times using a separatory funnel. DCM was then evaporated to yield the volatile *R. aromatica* residue, which was subsequently analyzed by GC-MS using the same method as described in Section 2.2.

### 4.4. Drying Methods of Plant Material

The plant material was collected using the same method as described in Section 2.1. The plant material was dried using three methods: shade-drying, oven-drying, and sun-drying. The shade-dry method involved storing plant material in a dark drawer at ambient temperature for 2 days, allowing all the leaves to dry. The oven-dry method was performed using a drying oven at 48 °C for 6 h, allowing the plant material to dry completely. The sun-drying method was performed by placing the plant material in the sun in mid-July 2023 and drying it for 2 days until completely dry. Each technique was then collected and distilled using the steam distillation method, as described in Section 2.2.

### 4.5. Solid-Phase Microextraction (SPME) Headspace Method

Three to six leaves of both *R. aromatica* and *R. trilobata* samples were added into an amber vial with distilled water, crushed to allow the volatile compounds to be released, and run through Solid-Phase Microextraction (SPME) headspace using a Shimadzu GC-2030 Gas Chromatograph machine, Shimadzu, Columbus, MD, USA coupled with a GCMS-TQ8050 NX Mass Spectrometer. For this specific experiment, the *R. trilobata* leaves were collected on 20 May 2023, in Battle Creek Canyon, Pleasant Grove, Utah 84062, USA (40°22′09.0″ N, 111°41′16.7″ W, 1828 m elevation).

## Figures and Tables

**Table 1:** The plant material and essential oil mass with the yield of each experiment.

Distillation	Date Collected	Plant Material Mass (g)	Essential Oil Mass (g)	% Yield
Week 1	22 May 2023	142.22	0.30	0.21
Week 2	30 May 2023	166.82	0.26	0.15
Week 3	14 June 2023	269.77	0.13	0.05
Week 4	16 June 2023	218.57	0.15	0.07
Flower	30 May 2023	157.24	0.55	0.35
Shade Dried	9 June 2023	104.89 (Dry Weight)	0.10	0.09
Oven Dried	11 July 2023	102.48 (Dry Weight)	0.31	0.31
Sun Dried	17 July 2023	142.02 (Dry Weight)	0.03	0.02

Yield expressed as % (*v*/*w*) on fresh-weight basis.

**Table 2:** Average temperature of each month the experiment was run.

Month	Average Temperature ^a^
May 2023	15 °C
June 2023	21 °C
July 2023	24 °C

^a^ Temperatures were taken from *Climate and Weather Averages in Pleasant Grove, Utah, USA*, n.d. https://www.timeanddate.com/weather/@5779816/climate accessed on 7 December 2025.

**Table 3:** GC-FID results showing essential oil percent composition of *R. aromatica*. Area % refers to GC-FID data.

No.	Compound	^a^ RI (Calc)	^b^ RI (Lit)	Week 1 (Area %)	Week 2 (Area %)	Week 3 (Area %)	Week 4 (Area %)	Flower (Area %)	Shade Dried (Area %)	Oven Dried (Area %)
1	Octane	801	800	-	-	-	-	tr	-	-
2	Heptanal	902	901	-	-	-	-	tr	-	-
3	Tricyclene	921	921	0.2	0.1	0.1	0.1	0.5	0.1	0.2
4	α-Thujene	924	924	tr	tr	-	-	tr	-	tr
5	α-Pinene	931	932	1.7	1.3	0.5	0.9	6.9	0.7	1.1
6	Camphene	948	946	0.6	0.5	0.3	0.4	2.2	0.3	0.6
7	Sabinene	972	969	0.2	0.6	-	tr	0.2	tr	0.1
8	β-Pinene	978	974	0.4	0.3	0.2	0.2	1.5	0.2	0.3
9	Myrcene	989	988	0.9	0.9	0.7	0.8	1.1	0.7	0.9
10	3-(*E*)-Hexenyl acetate	1001	1001	tr	0.1	0.1	0.1	-	-	tr
11	α-Phellandrene	1008	1002	1.8	1.5	1.3	1.5	3.5	1.4	1.8
12	α-Terpinene	1016	1014	tr	0.1	0.1	tr	tr	tr	0.1
13	*p*-Cymene	1025	1020	-	-	0.1	0.1	0.1	-	-
14	Limonene	1029	1024	87.2	85.8	85.5	86.3	76.5	79.6	77.6
15	β-Phellandrene	1030	1025	0.1	1.0	tr	0.1	1.1	0.2	0.3
16	(*Z*)-β-Ocimene	1034	1032	tr	tr	tr	tr	tr	tr	-
17	(*E*)-β-Ocimene	1044	1044	tr	tr	tr	tr	0.2	0.2	tr
18	γ-Terpinene	1058	1054	tr	0.1	0.1	0.1	0.1	0.1	0.1
19	Terpinolene	1086	1086	0.1	0.1	0.2	0.1	0.1	0.1	0.1
20	*p*-Cymenene	1091	1089	-	-	tr	-	-	tr	tr
21	Linalool	1099	1095	tr	tr	tr	tr	tr	tr	0.1
22	*n*-Nonanal	1105	1100	tr	tr	-	tr	0.1	tr	tr
23	2-Methyl-6-methylen-octa-1,7-dien-3-one	1112	1107	-	-	-	-	tr	-	-
24	Methyl octanoate	1122	1123	0.5	0.4	0.5	0.4	-	1.0	1.7
25	*trans*-Limonene oxide	1137	1137	tr	tr	-	-	-	-	-
26	Terpinen-4-ol	1180	1174	tr	0.1	-	-	tr	-	-
27	(3*Z*)-Hexenyl butanoate	1189	1184	tr	tr	0.1	tr	-	tr	-
28	α-Terpineol	1195	1186	tr	tr	0.1	tr	tr	-	tr
29	(3*Z*)-Hexenyl 2-methyl butanoate	1230	1229	-	-	-	-	-	tr	-
30	Carvone	1251	1239	tr	tr	tr	-	tr	tr	-
31	(2*E*)-Decenal	1263	1260	-	-	-	-	tr	-	-
32	Perilla aldehyde	1278	1269	tr	tr	-	tr	-	-	-
33	Vitispirane	1281	1286	-	-	-	-	-	-	tr
34	Bornyl acetate	1282	1287	0.3	0.3	0.5	0.4	0.5	0.5	0.6
35	Undecanal	1308	1305	tr	tr	-	-	tr	-	-
36	3-(*E*)-Hexenyl tiglate	1315	1315	-	-	-	-	-	tr	-
37	Methyl decanoate	1322	1323	tr	tr	tr	tr	-	0.1	0.1
38	δ-Elemene	1334	1335	tr	tr	-	-	-	-	-
39	α-Cubebene	1348	1345	tr	0.1	0.1	0.1	tr	0.1	0.1
40	Cyclosativene	1367	1369	-	-	-	-	-	-	0.1
41	α-Ylangene	1370	1375	tr	tr	0.1	tr	-	0.1	-
42	α-Copaene	1376	1374	0.1	0.1	0.3	0.2	0.1	0.4	0.3
43	β-Bourbonene	1382	1387	-	-	-	-	tr	tr	tr
44	β-Cubebene	1388	1387	tr	tr	tr	tr	tr	0.1	0.1
45	*trans*-β-Elemene	1391	1389	0.1	0.1	0.1	0.1	0.1	0.1	0.2
46	(*E*)-β-Caryophyllene	1418	1417	1.0	1.1	2.0	1.6	0.8	3.1	3.2
47	β-Copaene	1431	1430	tr	tr	0.1	0.1	tr	0.1	0.1
48	(*Z*)-β-Farnesene	1440	1440	-	-	-	-	tr	-	-
49	*cis*-Muurola-3,5-diene	1446	1448	tr	tr		-	-	-	tr
50	*trans*-Muurola-3,5-diene	1451	1451	tr	tr	tr	tr	-	0.1	0.1
51	α-Humulene	1454	1452	0.1	0.1	0.1	0.1	0.1	0.2	0.2
52	*cis*-Muuroladiene	1450	1452	-	-	tr	-	-	-	-
53	*cis*-Muurola-4(14),5-diene	1464	1465	-	-	tr	tr	-	tr	0.1
54	*cis*-Cadina-1(6),4-diene	1470	1461	0.1	tr	0.1	0.1	tr	0.2	0.1
55	α-Amorphene	1473	1483	-	-	0.2	-	-	0.3	-
56	*trans*-Cadina-1(6),4-diene	1479	1475	0.1	0.1	0.2	0.1	tr	0.2	0.3
57	Germacrene D	1484	1484	2.7	3.2	1.7	2.9	2.0	4.3	5.4
58	β-Selinene	1489	1489	-	-	tr	-	-	tr	tr
59	γ-Amorphene	1495	1495	-	-	0.1	-	-	-	-
60	*trans*-Muurola-4(14),5-diene	1492	1493	0.1	0.1	0.1	0.1	tr	0.2	0.2
61	Valencene	1494	1496	-	-	-	-	-	-	0.3
62	Viridiflorene	1496	1496	-	-	-	-	-	0.2	-
63	Bicyclogermacrene	1496	1500	0.1	0.1	0.1	0.1	0.1	-	-
64	Epizonarene	1500	1501	-	-	0.1	-	-	-	-
65	α-Muurolene	1502	1500	0.1	0.1	0.3	0.2	0.1	0.5	0.3
66	(*E,E*)-α-Farnesene	1504	1505	-	-	-	-	-	0.7	-
67	γ-Cadinene	1514	1513	0.1	0.1	0.1	0.1	tr	0.2	0.2
68	Cubebol	1516	1514	-	-	-	-	tr	-	-
69	δ-Cadinene	1520	1522	0.3	0.4	0.9	0.6	0.2	1.3	0.8
70	β-Sesquiphellandrene	1525	1521	-	-	-	-	tr	-	-
71	Zonarene	1527	1528	tr	tr	0.1	0.1	-	0.1	0.1
72	*trans*-Cadine-1,4-diene	1534	1533	tr	tr	0.1	tr	-	0.1	0.1
73	α-Cadinene	1537	1537	tr	tr	0.1	tr	-	0.1	0.1
74	α-Elemol	1549	1548	-	-	-	-	tr	-	-
75	(*E*)-Nerolidol	1561	1561	-	-	-	-	tr	-	-
76	10-*epi*-Juneol	1582	1579	-	-	-	tr	-	-	-
77	Germacrene D-4-ol	1581	1574	-	-	-	-	tr	-	-
78	Caryophyllene oxide	1583	1582	tr	tr	-	-	0.2	0.1	tr
79	Globulol	1584	1590	-	-	tr	tr	-	-	tr
80	Viridiflorol	1592	1592	-	-	-	-	-	-	tr
81	Khusimone	1603	1604	-	-	-	tr	-	tr	-
82	Guaiol	1593	1600	0.1	tr	0.1	0.1	0.2	0.1	0.1
83	1,10-di-*epi*-Cubenol	1616	1618	-	-	tr	tr	-	-	-
84	Humulane-1,6-dien-3-ol	1622	1619	tr	tr	-	0.1	-	0.1	-
85	1-*epi*-Cubenol	1626	1627	tr	tr	0.1	0.1	tr	0.1	0.1
86	*cis*-Cadin-4-en-7-ol	1634	1635	-	-	tr	tr	-	-	-
87	*epi*-α-Cadinol	1640	1638	0.1	0.1	0.3	0.1	0.1	0.2	0.2
88	*epi*-α-Muurolol	1642	1640	0.1	0.1	0.6	0.3	0.1	0.4	0.3
89	α-Muurolol	1643	1644	-	-	0.2	0.1	0.1	0.1	0.1
90	α-Cadinol	1655	1652	0.3	0.2	1.0	0.5	0.2	0.6	0.4
91	Bulnesol	1663	1670	-	-	0.1	tr	0.1	0.1	0.1
92	Mint sulfide	1735	1740	-	-	-	-	-	-	tr
93	Benzyl benzoate	1759	1759	-	-	-	-	tr	-	-
94	Phytone	1841	1845	-	-	-	-	tr	-	-
95	Pentacosane	2499	2500	0.1	tr	-	0.1	-	-	-
96	Heptacosane	2698	2700	0.1	0.1	-	0.1	-	-	-
	Total Identified			99.5	99.5	99.8	99.4	99.2	99.6	99.8

^a^ RI (calc) = Retention Index, determined with reference to a homologous series of normal alkanes on an ZB-5MS column. tr = trace amounts, <0.05% area. ^b^ RI (lit) = Retention Index from literature (see references).

**Table 4:** GC-MS results showing percent composition of *R. aromatica* hydrosols. *R. trilobata* hydrosol shown for comparison. Area % obtained through GC-MS.

No.	Compound	^a^ RI (Calc)	^b^ RI (Lit)	Week 1 (Area %)	Week 2 (Area %)	Week 3 (Area %)	Week 4 (Area %)	Shade Dried (Area %)	Oven Dried (Area %)	Sun Dried (Area %)	*R. trilobata* (Area %)
1	5-Methyl furfural	957	957	-	-	-	-	-	-	0.5	-
2	Hexanoic acid	967	967	-	-	0.1	0.1	-	-	0.6	-
3	Limonene	1029	1024	-	-	-	-	-	-	3.2	-
4	Benzene acetaldehyde	1036	1036	-	-	-	-	-	-	0.2	-
5	2-Acetyl pyrrole	1054	1054	-	-	-	-	-	-	0.7	-
6	*cis*-Linalool oxide (furanoid)	1067	1067	-	-	0.4	0.2	-	-	1.1	-
7	Pinol	1073	1083	-	-	0.2	-	-	-	0.2	-
8	*trans*-Linalool oxide (furanoid)	1087	1084	-	-	0.3	0.1	-	-	0.8	-
9	Linalool	1099	1095	-	1.1	0.9	3.8	-	-	0.4	-
10	Methyl octanoate	1122	1123	-	-	0.2	0.4	-	-	1.1	-
11	*cis-p*-Menth-2-en-1-ol	1125	1118	-	-	0.1	0.2	-	-	-	-
12	*trans-p*-Mentha-2,8-dien-1-ol	1122	1119	-	-	-	0.2	-	-	-	-
13	*trans-p*-Menth-2-en-1-ol	1142	1136	-	-	0.1	0.2	-	-	-	-
14	*cis*-β-Terpineol	1149	1140	-	-	2.6	1.3	-	-	-	-
15	Camphene hydrate	1156	1145	-	-	0.2	0.2	-	-	-	-
16	*trans*-β-Terpineol	1156	1159	-	-	2.0	2.2	-	-	-	-
17	*p*-Mentha-1,5-dien-8-ol	1171	1166	-	-	2.2	-	-	-	2.8	-
18	Octanoic acid	1171	1167	6.1	2.8	11.5	-	1.8	48.6	14.5	0.9
19	Terpinen-4-ol	1180	1174	3.0	4.7	2.5	12.8	-	-	7.9	-
20	*p*-Cymen-8-ol	1187	1179	-	-	0.5	0.4	-	-	0.5	-
21	Ethyl octanoate	1196	1196	-	-	-	-	-	-	0.5	-
22	α-Terpineol	1195	1186	23.2	4.2	37.3	21.3	-	3.8	7.6	3.7
23	Verbenone	1206	1204	-	-	0.1	-	-	-	-	-
24	Borneol	1171	1165	-	-	-	-	-	-	2.6	-
25	*p*-Ethoxystyrene	1150	-	-	-	0.2	0.2	-	0.6	0.7	-
26	Cryptone	1187	1183	-	-	-	-	-	-	3.0	-
27	Safranal	1195	1196	-	-	-	-	-	-	0.3	-
28	Carvone	1251	1239	-	-	1.6	1.8	-	-	1.3	-
29	*trans*-Carveol	1218	1215	-	-	5.4	2.4	-	-	-	-
30	Carvotanacetone	1248	1244	-	-	0.1	-	-	-	0.3	-
31	Nonanoic acid	1267	1267	-	-	-	-	-	-	0.2	0.3
32	*iso*-Piperitenone	1270	**1272**	-	-	0.2	0.2	-	-	-	-
33	Perilla aldehyde	1278	1269	-	-	0.6	1.0	-	-	0.4	-
34	Bornyl acetate	1282	1287	-	0.4	0.8	2.0	-	-	3.9	-
35	Thymol	1289	1289	-	-	0.2	0.4	-	1.1	-	0.6
36	*p*-Vinylguaiacol	1309	1309	-	-	0.7	7.6	-	0.1	-	-
37	Carvacrol	1297	1298	-	-	-	-	-	-	0.7	-
38	Methyl decanoate	1323	1323	-	-	-	-	-	-	0.8	-
39	α-Cubebene	1348	1345	-	-	-	-	-	-	0.2	-
40	Eugenol	1357	1356	-	-	0.2	0.2	10.5	0.1	0.6	0.6
41	α-Copaene	1376	1374	-	-	-	-	-	-	1.0	-
42	Decanoic acid	1368	1364	-	-	-	0.1	-	-	-	-
43	Methyl perillate	1387	1392	-	-	-	-	-	0.2	-	-
44	(*Z*)-Jasmone	1389	1392	-	0.5	1.1	1.0	-	-	0.3	0.1
45	Tetradecane+Vanillin	1400 + 1398	1400 + 1393	-	-	-	-	-	-	0.3	-
46	(*E*)-Jasmone	1390	1390	3.8	-	-	-	-	0.3	-	-
47	Perillic acid	1467	-	-	-	-	-	-	0.6	-	-
48	Coumarin	1434	1432	-	-	-	-	4.4	-	-	-
49	Tetradecane	1400	1400	-	0.4	0.2	0.5	-	-	-	-
50	β-Caryophyllene	1418	1417	-	-	-	0.6	-	-	9.2	0.8
51	Florhydral	1424	-	-	-	-	-	-	-	0.3	-
52	β-Copaene	1431	1430	-	-	-	-	-	-	0.2	-
53	Geranyl acetone	1448	1453	-	-	-	-	-	-	0.8	-
54	Perillyl acetate	1433	1436	-	-	0.1	0.2	-	-	-	-
55	α-Humulene	1454	1452	-	-	-	-	-	-	0.7	0.2
56	*cis*-Cadina-1(6),4-diene	1470	1461	-	-	-	-	-	-	0.2	-
57	*trans*-Cadina-1(6),4-diene	1473	1475	-	-	-	0.2	-	-	1.0	0.2
58	α-Amorphene	1479	1483	-	-	-	-	-	-	0.5	0.2
59	(*E*)-*iso*-Eugenol	1444	1448	-	-	0.2	-	-	0.7	0.5	-
60	Germacrene D	1484	1484	-	-	0.1	2.4	-	0.5	13.2	2.2
61	Jasmolactone	1487	-	-	-	-	-	-	0.2	-	-
62	6-*epi*-Shyobunone	1488	1481	-	-	-	-	-	-	-	0.4
63	Viridiflorene	1490	1496	-	-	-	-	-	-	-	0.2
64	Bicyclogermacrene	1496	1500	-	-	-	-	-	-	-	0.3
65	α-Muurolene	1500	1500	-	-	-	0.3	-	-	1.0	0.8
66	Shyobunone	1508	1503	-	-	-	-	-	-	-	1.1
67	γ-Cadinene	1514	1513	-	-	-	0.1	-	-	0.6	0.5
68	δ-Cadinene	1519	1522	-	-	0.2	1.1	-	0.3	2.6	3.1
69	Zonarene	1523	1528	-	-	-	-	-	-	-	0.3
70	*trans*-Cadine-1,4-diene	1532	1533	-	-	-	-	-	-	0.3	0.1
71	α-Cadinene	1537	1537	-	-	-	-	-	-	0.2	0.2
72	(*E*)-Nerolidol	1561	1561	-	-	-	-	-	-	-	1.3
73	Dihydro actinidiolide	1524	1519	-	-	-	-	-	-	0.3	-
74	Caryophyllenyl alcohol	1570	1570	-	-	-	-	-	1.1	-	2.0
75	Caryophyllene oxide	1583	1582	-	-	-	-	-	0.2	0.2	-
76	Guaiol	1593	1600	-	-	-	-	-	1.7	-	-
77	Hexadecane	1600	1600	6.0	1.0	-	-	1.4	-	-	-
78	Globulol	1590	1582	-	-	0.2	0.4	-	-	-	2.0
79	Cubeban-11-ol	1596	1595	-	-	-	-	-	-	-	1.2
80	Viridiflorol	1592	1592	-	-	0.3	0.2	-	0.5	-	-
81	Hexadecane + Khusimone	1600 + 1604	1600 + 1604	-	-	-	-	-	0.3	-	-
82	1,10-di-*epi*-Cubenol	1616	1618	-	-	0.2	0.2	-	0.3	-	0.3
83	Humulane-1,6-dien-3-ol	1623	1757	-	-	0.5	0.6	0.5	-	-	-
84	1-*epi*-Cubenol	1626	1627	2.0	0.4	1.0	1.5	2.0	1.5	0.3	2.5
85	γ-Eudesmol	1631	1630	-	-	-	-	-	0.4	-	-
86	Methyl (*Z*)-jasmonate	1649	1648	-	-	-	-	1.7	0.1	-	-
87	Cubenol	1641	1645	-	-	-	-	-	0.6	-	-
88	*epi*-α-Cadinol	1640	1638	-	1.1	1.9	2.6	4.3	3.4	1.1	6.6
89	*epi*-α-Muurolol	1642	1640	16.7	3.9	4.9	5.5	26.1	6.4	2.0	11.8
90	α-Muurolol	1643	1644	-	1.4	3.7	5.6	-	3.5	1.2	5.3
91	α-Cadinol	1655	1652	33.4	8.8	12.0	14.2	41.9	16.3	4.6	31.0
92	Selin-11-en-4α-ol	1659	1658	-	-	-	-	-	0.3	-	-
93	Bulnesol	1663	1670	5.8	-	0.5	0.8	-	1.2	-	-
94	Octadecane	1800	1800	-	2.9	-	-	0.6	-	-	-
95	Juniper camphor	1699	1700	-	-	-	-	-	-	-	0.5
96	*iso*-Calamenediol	1744	1760	-	-	-	-	-	-	-	3.7
97	Myristic acid	1758	1769	-	-	-	-	-	-	-	0.5
98	(*Z*)-Thujopsenic acid	1827	1863	-	-	0.2	-	-	-	-	-
99	Palmitic acid	1958	1963	-	0.9	-	-	0.5	-	-	3.1
100	Linoleic acid	2120	2126	-	-	-	-	-	-	-	0.4
101	Stearic acid	2126	2170	-	0.4	-	-	-	-	-	-
102	Eicosane	2006	2000	-	4.5	-	-	-	-	-	-
103	*iso*-Linoleic acid	2188	2192	-	-	-	-	-	1.1	-	-
104	(*Z*)-9-Tricosene	2267	2315	-	-	-	-	-	0.9	-	-
105	Docosane	2200	2200	-	5.1	-	-	-	-	-	-
106	Tricosane	2300	2300	-	6.2	-	-	-	-	-	-
107	Tetracosane	2401	2400		5.3	-	-	-	-	-	-
108	Pentacosane	2499	2500	-	-	-	-	-	-	-	0.3
109	Hexacosane	2600	2600	-	5.7	-	-	-	-	-	-
110	Heptacosane	2698	2700	-	-	-	0.3	1.1	0.1	-	1.4
111	Octacosane	2791	2800	-	5.9	-	-	-	-	-	0.2
112	Nonacosane	2899	2900	-	-	-	0.3	0.9	0.1	-	2.2
113	Dotriacontane	3200	3200	-	11.4	-	-	-	-	-	-
114	Tetratriacontane	3400	3400	-	11.9	-	-	-	-	-	-
115	Hexatriacontane	3593	3600	-	3.8	-	-	-	-	-	-
116	Octatriacontane	3793	3800	-	3.0	-	-	-	-	-	-
	Total Identified			100.00	100.0	97.6	98.2	97.5	98.4	99.5	93.0

^a^ RI (calc) = Retention Index, determined with reference to a homologous series of normal alkanes on a ZB-5MS column. ^b^ RI (lit) = Retention Index from literature (see references). Yields for drying treatments are expressed on a dry-weight basis.

**Table 5:** SPME headspace to compare *Rhus aromatica* and *Rhus trilobata*.

No.	Compound	^a^ RI (Calc)	^b^ RI (Lit)	*R. aromatica* (Area %)	*R. trilobata* (Area %)
1	α-Pinene	931	932	1.2	22.3
2	Camphene	948	946	0.1	-
3	Sabinene	972	969	-	6.0
4	Myrcene	989	988	1.0	2.0
5	3-cis-Hexenyl acetate	1005	-	-	0.1
6	Hexenyl acetate+Pseudolimonene	1005 + 1003	1003	3.7	-
7	δ-3-Carene	1008	1008	-	0.3
8	Limonene	1029	1024	59.2	2.0
9	(*Z*)-β-Ocimene	1034	1032	-	6.4
10	(*E*)-β-Ocimene	1044	1044	-	1.2
11	γ-Terpinene	1058	1054	0.1	1.8
12	Terpinolene	1086	1086	0.3	0.9
13	(3*Z*)-Hexenyl propionate+Linalool	1095 + 1099	1095 + 1095	0.1	0.4
14	2-Methyl-6-methylen-octa-1,7-dien-3-one	1112	-	-	0.2
15	Methyl octanoate	1122	1123	0.1	0.4
16	*allo*-Ocimene	1128	1128	-	4.3
17	*trans*-Limonene oxide	1137	1137	0.4	-
18	(3*Z*)-Hexenyl isobutanoate	1142	1142	0.1	0.3
19	Terpinen-4-ol	1180	1174	tr	2.9
20	(3*Z*)-Hexenyl butanoate	1189	1184	0.7	3.5
21	Dihydro carvone	1205	1200	tr	-
22	*trans*-Carveol	1218	1215	0.4	-
23	(2*E*)-Hexenyl butanoate	1193	1193	-	1.3
24	(3*Z*)-Hexenyl 2-methyl butanoate	1230	1229	0.1	1.8
25	Perilla aldehyde	1278	1269	0.1	-
26	Bornyl acetate	1282	1287	0.5	-
27	Limonen-10-ol	1288	1288	0.3	-
28	Limonene dioxide	1085	-	0.1	-
29	2-cis-Hexenyl isovalerate	1241	1241	-	0.8
30	Perilla alcohol	1294	1294	0.5	-
31	3-trans-Hexenyl tiglate	1315	1315	-	0.2
32	Bicycloelemene	1321	-	0.2	-
33	δ-Elemene	1334	1335	-	1.1
34	α-Cubebene	1348	1345	0.4	0.2
35	Cyclosativene	1367	1369	-	0.2
36	α-Ylangene	1370	1375	0.1	0.1
37	α-Copaene	1376	1374	1.3	-
38	(3*Z*)-Hexenyl hexanoate+α-Copaene	1378 + 1376	1378 + 1374	-	1.6
39	Hexyl hexanoate	1382	1382	-	0.1
40	(2*E*)-Hexenyl caproate	1385	1385	-	0.3
41	Unidentified	-	-	1.1	-
42	β-Cubebene	1388	1387	-	0.4
43	*trans-β*-Elemene	1391	1389	-	0.4
44	(*E*)-β-Caryophyllene	1418	1417	5.9	9.4
45	β-Copaene	1431	1430	1.5	1.6
46	*cis*-Muurola-3,5-diene	1446	1448	0.2	0.2
49	*trans*-Muurola-3,5-diene	1451	1451	0.2	0.2
50	Unidentified	-	-	1.2	-
51	α-Humulene	1454	1452	-	1.4
52	*cis*-Muurola-4(14),5-diene	1464	1465	0.9	0.9
53	*cis*-Cadina-1(6),4-diene	1470	1461	0.1	0.1
54	*trans*-Cadina-1(6),4-diene	1473	1475	0.3	0.3
55	Germacrene D	1484	1484	12.0	13.4
56	*trans*-Muurola-4(14),5-diene	1492	1493	0.8	0.7
57	α-Muurolene	1502	1500	1.4	-
58	Bicyclogermacrene	1496	1500	-	2.6
59	Trans, trans-α-Farnesene	1504	1505	-	1.0
61	γ-Cadinene	1514	1513	0.6	0.5
62	δ-Cadinene	1519	1522	0.1	1.4
63	*trans*-Cadine-1,4-diene	1534	1533	0.1	0.1
64	α-Cadinene	1537	1537	0.3	0.2
65	1,5-Epoxy salvial-4(14)-ene+(3*Z*)-Hexenyl benzoate	1566 + 1572	-+1565	tr	-
66	Guaiol	1593	1600	tr	-
67	(*E*)-Nerolidol	1561	1561	-	0.1
68	(3*Z*)-Hexenyl benzoate	1572	1565	-	0.2
69	*cis*-Dihydro apofarnesol	1571	-	-	0.1
70	*epi*-α-Cadinol	1640	1638	0.1	0.2
71	δ-Cadinol	1644	-	tr	-
72	α-Cadinol	1655	1652	0.1	0.2
	Total Identified			95.6	98.1

^a^ RI (calc) = Retention Index, determined with reference to a homologous series of normal alkanes on an ZB-5MS column. tr = trace amounts, <0.05% area. ^b^ RI (lit) = Retention Index from literature (see references).

**Table 6:** GC Chiral results showing the enantiomers of some of the compounds found in *R. aromatica*—enantiomeric distribution.

Compound	Week 1 (%)	Week 2 (%)	Week 3 (%)	Week 4 (%)	Flower (%)	Shade Dried (%)	Oven Dried (%)
(+)-α-Pinene	67.52	67.70	64.76	70.78	60.64	67.95	67.91
(-)-α-Pinene	32.48	32.30	35.24	29.22	39.36	32.05	32.09
(+)-Camphene	4.43	4.33	5.61	4.79	4.20	5.25	5.09
(-)-Camphene	95.57	95.67	94.39	95.21	95.80	94.75	94.91
(+)-Sabinene	100.00	100	-	-	100.00	-	100.00
(-)-Sabinene	-	-	-	-	-	-	-
(+)-β-Pinene	9.60	9.31	8.34	7.84	7.78	9.87	9.59
(-)-β-Pinene	90.40	90.69	91.66	92.16	92.22	90.13	90.41
(+)-α-Phellandrene	99.71	99.53	100.00	100.00	99.71	100.00	100.00
(-)-α-Phellandrene	0.29	0.47	-	-	0.29	-	-
(+)-Limonene	99.07	99.12	100.00	100.00	98.53	100.00	100.00
(-)-Limonene	0.93	0.88	-	-	1.47	-	-
(+)-β-Phellandrene	100.00	67.86	-	-	65.82	-	-
(-)-β-Phellandrene	-	32.14	-	-	34.18	-	-
(-)-Bornyl acetate	100.00	100.00	100.00	100.00	100.00	100.00	100.00
(-)-α-Copaene	-	-	100.00	-	-	100.00	100.00
(-)-β-Caryophyllene	100.00	100.00	100.00	100.00	100.00	100.00	100.00
(-)-Germacrene D	100.00	100.00	100.00	100.00	100.00	100.00	100.00
(+)-δ-Cadinene	100.00	100.00	100.00	100.00	100.00	100.00	100.00

Enantiomeric distribution where D enantiomer is (+), and L enantiomer is (-).

## Data Availability

All data are available in the manuscript.
